# Trophic Niche Differentiation in Two Sympatric Nuthatch Species (*Sitta yunnanensis* and *Sitta nagaensis*)

**DOI:** 10.3390/ani14081146

**Published:** 2024-04-10

**Authors:** Qiang Guo, Xi Lu, Chongxin Xie, Jiansong Zhang, Xianyin Xu, Yuhan Qian, Xu Luo, Yubao Duan

**Affiliations:** 1 Key Laboratory for Forest Resources Conservation and Utilization in the Southwest Mountains of China, Ministry of Education, Southwest Forestry University, Kunming 650224, China; guoq9382@163.com (Q.G.); lx95212024@163.com (X.L.); jiansong0311@163.com (J.Z.); 2 College of Forestry, Southwest Forestry University, Kunming 650224, China; chongxinxie@163.com (C.X.); nerv6667@163.com (Y.Q.); 3 Key Laboratory for Conserving Wildlife with Small Populations in Yunnan, Southwest Forestry University, Kunming 650224, China; 4 Administration of Zixi Mountain Provincial Nature Reserve, Chuxiong 675008, China

**Keywords:** niche differentiation hypothesis, stable isotope, *Sitta nagaensis*, sympatric species, *Sitta yunnanensis*, trophic niche

## Abstract

**Simple Summary:**

Trophic niches, as one of the important dimensions of niche theory, show the nutritional requirements of species; they can reflect their position in the ecosystem trophic level, and functional status, as well as the nutritional relationships between species. Similarities in body structure and ecological needs may lead to interspecific nutritional competition, and species may undergo niche differentiation through different foraging strategies. The body tissues of consumers contain stable isotope signatures that reflect their dietary information, and stable isotope analysis has been widely used in animal diet research. In this study, stable isotope techniques were used to assess the dietary information and nutritional niches of *Sitta yunnanensis* and *Sitta nagaensis* coexisting in Yunnan Province. *S. yunnanensis* and *S. nagaensis* mainly preyed on six orders, including Orthoptera, with overlapping food resources but different dietary ratios. Their two niches were differentiated, with *S. yunnanensis* having wider trophic niches. This study provides new insights into the food resources and trophic niches of *S. yunnanensis* and *S. nagaensis*, and the mechanisms behind their coexistence were consistent with the niche hypothesis.

**Abstract:**

Sympatric closely related species may experience interspecific trophic competition due to ecological similarity; they may isolate in terms of diet or habitat use as a strategy to avoid competition. The body tissues of consumers contain stable isotope signatures information that can be applied to infer their dietary information. In this study, δ^13^C and δ^15^N stable isotopes were analyzed to determine the dietary information and trophic niches of sympatric coexisting *Sitta yunnanensis* and *Sitta nagaensis*. The results showed that the food sources of *S. yunnanensis* and *S. nagaensis* were from six orders, including Orthoptera, and the cumulative contribution rate was 99.97%, with the two species eating similar diets but at different rates. The larger δ^13^C of *S. yunnanensis* indicates that it had a wider range of habitats for feeding, while the difference in δ^15^N values was not significant (*p* > 0.05), indicating that both species feed on similar nutrient levels. As determined by Bayesian ellipses, the isotopic niches of *S. yunnanensis* and *S. nagaensis* were differentiated; the isotopic niche width of *S. yunnanensis* is 2.69‰^2^, which was larger than that of *S. nagaensis* (0.73‰^2^), indicates that differentiation between the two species in diet or habitat use reduced competition. Trophic niche differentiation and differences in foraging proportions may be the principal resource allocation mechanisms behind *S. yunnanensis* and *S. nagaensis* coexistence.

## 1. Introduction

The coexistence of closely related sympatric species has been a core topic in the maintenance of biodiversity and community ecology [1,2,3]. The close phylogenetic relationships between closely related species often result in morphological similarities, leading to similar dietary strategies and ecological requirements [4]. For sympatric closely related species with similar ecological requirements, stable coexistence can only be achieved by reducing overlap in the use of limited resources [5]. At present, there are many theories or hypotheses existing to explain the mechanism behind species coexistence, among which the niche differentiation hypothesis is the most prevailing [6,7]. The niche differentiation hypothesis states that the body structure, behavior, and resource needs of related sympatric species are very similar, and niche separation is conducive to reducing competition, playing an important role in stable coexistence [8,9]. Two species with completely overlapping ecological niches cannot co-exist in the same domain for a long time [10]. Differentiation in at least one dimension is needed to achieve stable co-existence, such as temporal or trophic niche differentiation [11,12,13,14]. Sympatric species may achieve trophic niche separation by foraging for different foods, at different places, or at different times [15]. As one of the important dimensions of niche theory, the study of trophic niches is very important for understanding the coexistence mechanisms and ecology of closely related species to improve conservation strategies [16].

In birds, trophic niche studies usually use direct observation [17] and gastrointestinal content analysis [18]; these methods require substantial time and manpower, conflict with non-invasive studies of endangered wildlife, and can only identify a small percentage of food resources [4,19]. Compared to traditional methods, stable isotope analysis overcomes these limitations to evaluate the dietary composition of consumers using tissue synthesis based on the metabolic activity on different time scales [19,20]. In birds, feathers remain metabolically inert, and keratin in feathers stops isotope fractionation; because of this, the isotopic information recorded in feather tissue reflects the dietary composition during molting the previous year [21,22]. In addition, isotopic niches can be calculated based on different elements. δ^13^C changes little in the process of food chain transmission and is often used to represent food sources and habitat, while δ^15^N is highly enriched between trophic levels and is commonly used to evaluate the trophic level of the consumer; the combination of δ^13^C and δ^15^N can be used to analyze consumers’ consumption of food resources [23,24,25].

The Yunnan Nuthatch (*Sitta yunnanensis*) and Chestnut-vented Nuthatch (*S. nagaensis*) are closely related species with similar morphology, including plumage pattern, body size, and bill shape [26]. *S. yunnanensis* and *S. nagaensis* inhabit coniferous and mixed-coniferous forests at altitudes of 1500–3000 m [27]. Both species inhabit the same habitat and feed on insects on tree trunks in the Zixishan Provincial Nature Reserve, Yunnan Province. Due to their similar morphology and eating habits and their stable co-existence in the study area, they likely share resources. The main goal of this study was to understand the feeding ecology of *S. yunnanensis* and *S. nagaensis* to evaluate their trophic niches and food resource partitioning. Feathers were collected from *S. yunnanensis* and *S. nagaensis*, along with potential food samples and differences in their δ^13^C and δ^15^N values, which were examined to analyze their feeding ecology. It was hypothesized that *S. yunnanensis* and *S. nagaensis* both feed on similar food resources but at different proportions, which would be reflected in the δ^13^C and δ^15^N values; they were expected to have differentiated trophic niche, which would be reflected in their isotopic niche overlaps.

## 2. Materials and Methods

### 2.1. Study Area

The Zixi Mountain Provincial Nature Reserve of Yunnan Province is located southwest of Chuxiong City, Yunnan Province, China at 101°22′–101°26′ E and 24°58′–25°04′ N, with an altitude of 1950–2502 m and a total area of 160 km^2^ [27]. The reserve is a narrow strip of hilly land running from north to south with east and west slopes, most of which is located on the upper part of the mountain. The highest point is the main peak of Zixi Mountain, and the lowest point is the exit of the Maji River. The reserve area is located in the monsoon belt of the northern subtropical plateau, and experiences significant seasonal changes; the summer has abundant rainfall and high temperatures, while the winter is relatively dry with low temperatures. The annual average precipitation is 900 mm, with a rainy season from May to October and a dry season from November to April. The average annual temperature is 12.1–14.9 °C [27]. The vegetation types in the protected area are secondary coniferous and mixed coniferous forests. The forest types are predominantly Yunnan and Huashan Pine forests.

### 2.2. Sample Collection

#### 2.2.1. Bird Feather Collection

In this study, feathers from *S. yunnanensis* and *S. nagaensis* were collected in the study area from December 2018 to May 2019. Feathers from 13 individuals of *S. nagaensis* and 22 individuals of *S. Yunnanensis* were collected (including five young *S. nagaensis*). The mist nets used collects about 1 cm^2^ cm from the outermost tip of the blade of each adult bird’s tenth primary feather (P10) before releasing it [28]. In addition, the feathers of young birds (about 16 days old) were collected. To avoid influencing feather regeneration in nestlings, the feathers collected were clipped 1 cm from the feather tip and put into a Ziploc bag for storage in a portable refrigerator. After sampling, adult birds were promptly released, and nestlings were promptly put back into their nest. Samples were brought back to the laboratory and refrigerated at −20 °C for subsequent analyses [29].

#### 2.2.2. Food Source Sample Collection

During the rearing period of *S. yunnanensis* and *S. nagaensis*, two methods were used to determine food sources without disrupting normal activities. For *S. nagaensis*, a digital camera (7D2, Canon, Japan) was used to take high-resolution photographs of birds during feeding at a distance of 10 m from the nest cavity entrance. Based on morphological characteristics, the photos were used to identify food items to the order level by referring to the Chinese Insect Atlas [30], the Chinese Insect Ecology Atlas [31], and the Insect Family Tree [32].

Based on photos and field observations, potential food insect samples were collected by net trapping in the area near the breeding nests of the two species. After the insects were collected, they were sealed in a collection bottle or Ziplock bag and stored in a portable refrigerated box. They were taken to the laboratory for identification and used for stable isotope analyses [29].

### 2.3. Sample Processing and Testing

The feather samples were taken to the laboratory to remove impurities. Since degreasing would affect the nitrogen isotope value, the treated feather samples were divided into two parts: one was degreased to determine the stable carbon isotope value and the other was used directly to determine the stable nitrogen isotope value [33]. All samples were sent to ShenZhen Huake JingXin Detection Technology Company for testing the stable isotope ratios of carbon and nitrogen using a DELTA V Advantage Isotope Ratio Mass Spectrometer (Isotope Ratio Mass Spectrometer, Thermo Fisher Scientific, Inc., Bremen, Germany) and EA-HT Element analyzer (Thermo Fisher Scientific, Inc., Bremen, Germany).

### 2.4. Data Processing

Accuracy was ≤0.5% for C%, ≤0.1‰ for δ13C, ≤0.5% for N%, and ≤0.2‰ for δ15N. The natural abundance of stable carbon and nitrogen isotopes (enrichment) was expressed as follows:

δX = [(R_sample_/R_standard_) − 1] × 10^3^

where δX is δ^13^C or δ^15^N, R_sample_ is the isotope ratio ^13^C/^12^C or ^15^N/^14^N of the measured sample, and R_standard_ is the ^13^C/^12^C or ^15^N/^14^N ratio of the reference material. The δ^13^C value was reported relative to the international reference material PDB (R scale = 0.0112372) and the δ^15^N value was relative to standard atmospheric nitrogen (R scale = 0.0036765).

The Bayesian stable isotope mixing model in the R package “MixSiar” was used to calculate the dietary contributions of different food resources for both species [4,34]. The average δ^13^C and δ^15^N values of the food source samples, and the δ^13^C and δ^15^N values of the *S. yunnanensis* and *S. nagaensis* feathers were input into the R package “SIAR”. After applying nutrient enrichment factors for carbon and nitrogen, the model was fitted to determine the relative contributions of various food sources [35]. The isotopic niches of *S. yunnanensis* and *S. nagaensis* were calculated using stable isotope Bayes ellipses in the R package “SIBER” [35]. The total convex hull area (TA) and corrected standard elliptic area (SEAc) were used to quantify isotopic niche width and calculate their overlap [35]. The enrichment factors for carbon and nitrogen stable isotopes could not be obtained in the laboratory owing to limitations in the experimental conditions. Accordingly, estimates from a meta-analysis by Post et al. (2002) were used (Δ^13^C = 0.4 ± 1.3‰ and Δ^15^N = 3.4 ± 1‰); these values are widely applicable to most ecosystems [36].

Statistical analyses were performed using Excel 2010 and R (Version 4.2.2). The stable isotope values (δ^13^C and δ^15^N) of different food sources and different bird samples were compared by one-way analysis of variance. All isotopic data are expressed as mean ± SD, and *p* < 0.05 was considered significant [35].

## 3. Results

### 3.1. Stable Isotope Characteristics of Food Sources

Insects belonging to six orders were identified in 45 out of a total of 144 photographs of birds during feeding. Diptera, Orthoptera, and Lepidoptera were most frequently observed. Both *S. yunnanensis* and *S. nagaensis* consumed insects belonging to all six orders; however, the number of insects was not recorded for *S. nagaensis*.

Six items from 47 foods were collected in the study area. The carbon (C‰) content of these six potential food sources ranged from 47.24‰ to 69.48‰, while the nitrogen (N‰) content ranged from 3.94‰ to 12.50‰ (Table S1). The δ^13^C values ranged from −23.98‰ to −29.18‰, with the highest values for Orthoptera and the lowest values for Lepidoptera. The δ^15^N values ranged from 0.90‰ to 3.97‰, with the highest values for Coleoptera at 3.97‰ and the lowest values for Orthoptera at 0.90‰ (Table 1).

### 3.2. Stable Isotope Results for S. yunnanensis and S. nagaensis Samples

**Figure 1:**
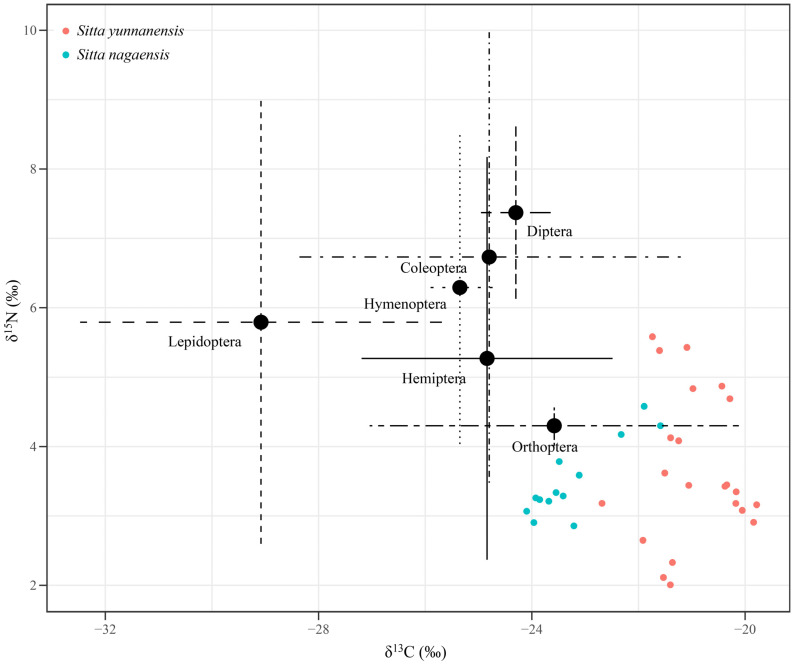
Stable isotope biplot of δ^13^C and δ^15^N values for *S. yunnanensis* and *S. nagaensis* feathers at the Yunnan Zixi Mountain and potential food sources.

The δ^13^C values for *S. yunnanensis* feather samples (*n* = 22) ranged from −22.68‰ to −19.78‰, with an average of −20.95 ± 0.77‰; the δ^15^N values ranged from 2.01‰ to 5.58‰, with an average value of 3.67 ± 1.07‰ (Figure 1 and Table S2). Since there were no significant differences in δ^13^C and δ^15^N between features of adult birds (*n* = 8) and baby birds (*n* = 5) (*p* > 0.05), an integrated analysis showed that δ^13^C values of *S. nagaensis* feathers (*n* = 13) ranged from −24.10‰ to −21.59‰, with an average of −23.24 ± 0.81‰, while δ^15^N values ranged from 2.86‰ to 4.58‰, with an average value of 3.50 ± 0.54‰ (Figure 1 and Table S2).

The δ^13^C values of *S. yunnanensis* and *S. nagaensis* feathers were significantly different (*p* < 0.001), indicating that food resources were differentiated and feeding niches were separated. There was no significant difference in δ^15^N values (*p* = 0.59), indicating that the species occupied the same trophic position (Figure 1).

### 3.3. Food Source Composition from S. yunnanensis and S. nagaensis Feathers

**Figure 2:**
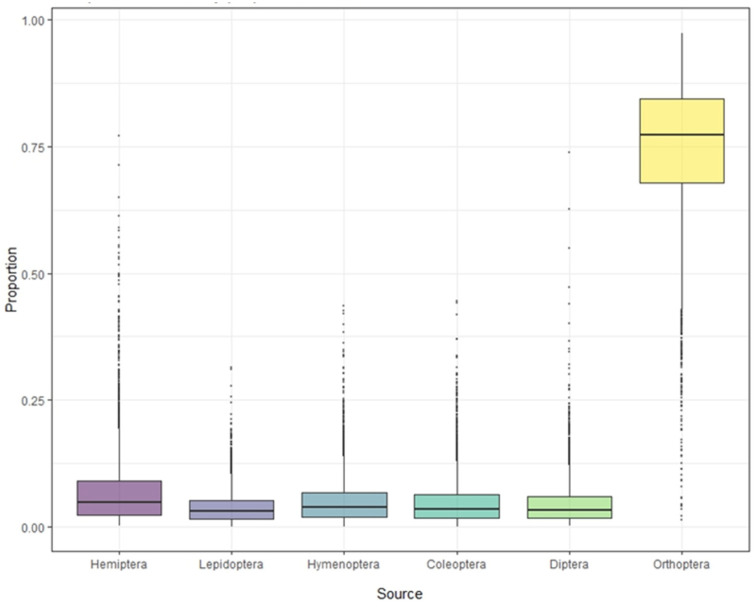
Contribution of each food source to the diet of *S. yunnanensis*, based on stable isotope analyses.

**Figure 3:**
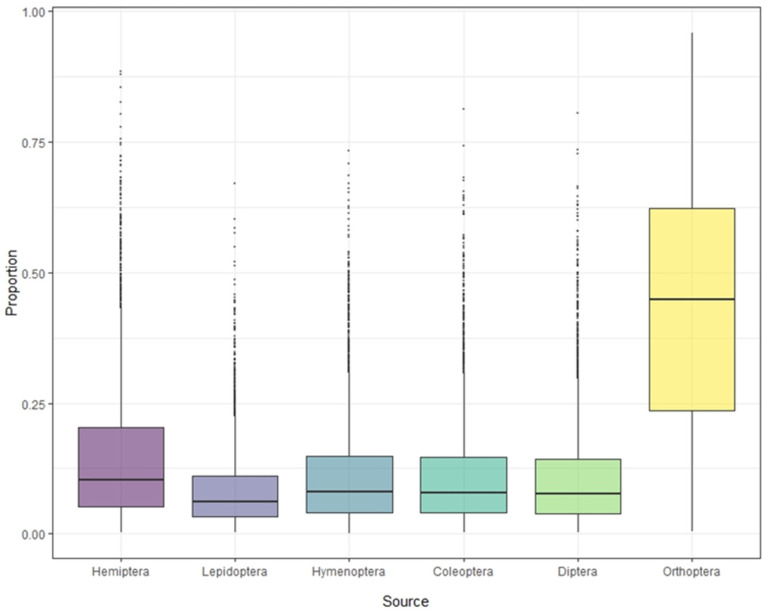
Contribution of each food source to the diet of *S. nagaensis*, based on stable isotope analyses.

Stable isotope analysis of feathers of *S. yunnanensis* and *S. nagaensis* showed that the cumulative contribution rate of insects in the six orders to their diet reached 99.97% (Table 1). Although not all food species were collected, the results effectively reflect the composition and contribution rate of the main food sources. The contribution rates of food sources in feather samples from *S. yunnanensis* were as follows: Orthoptera > Hemiptera > Coleoptera > Hymenoptera > Diptera > Lepidoptera; for *S. nagaensis* the order was Orthoptera > Hemiptera > Hymenoptera > Coleoptera > Diptera > Lepidoptera. The contribution rate of Orthoptera to the diet of *S. yunnanensis* based on feather samples was as high as 78.69%, while its contribution to the diet of *S. nagaensis* was 45.56%, indicating that it was an important food source for both species (Figure 2 and Figure 3, Table 1). Lepidoptera had the lowest dietary contribution rates 2.29% for *S. yunnanensis* and 4.12% for *S. nagaensis* (Figure 2 and Figure 3, Table 1).

### 3.4. Niche Relationships Reflected by Feather Samples from S. yunnanensis and S. nagaensis

**Figure 4:**
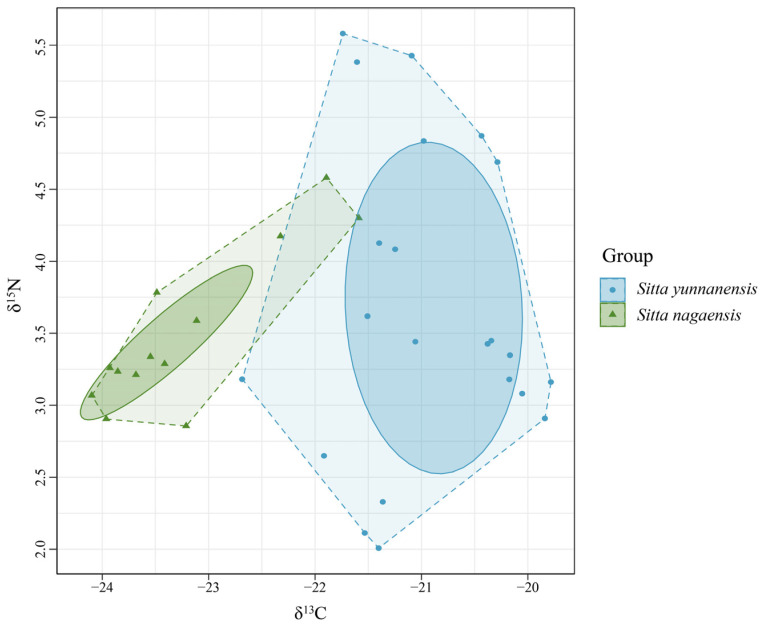
Isotopic niches of *S. yunnanensis* and *S. nagaensis*.

Bayes standard ellipses of the trophic niches of *S. yunnanensis* and *S. nagaensis* were fitted with δ^15^N as the horizontal coordinate and δ^13^C as the vertical coordinate (Figure 4). The total vegetative niche area (TA) and correctional ellipse area (SEAc) of *S. yunnanensis* were both larger than those of *S. nagaensis*, and there was no overlap between the correctional ellipses, indicating that there was differentiation between the niches of the two species. The niche width of *S. yunnanensis* (2.69‰^2^) was larger than that of *S. nagaensis* (0.73‰^2^), indicating that *S. yunnanensis* had a wider feeding range (Table 2, Figure 4).

## 4. Discussion

In this study, new information on the trophic ecology of the sympatric coexistence of *S. yunnanensis* and *S. nagaensis* was identified. As hypothesized, stable isotope analysis showed that *S. yunnanensis* and *S. nagaensis* shared similar diets and trophic niche differentiation within the study area, though *S. yunnanensis* (2.69‰^2^) had a wider ecological niche than *S. nagaensis* (0.73‰^2^).

Orthoptera, Hemiptera, Hymenoptera, Coleoptera, Diptera, and Lepidoptera were found to be common food source of *S. yunnanensis* and *S. nagaensis* (Table 1 and Figure 2 and Figure 3), as the cumulative contribution rate of the six orders of insects to the diets of both species reached 99.97% (Table 1). This was consistent with field observations showing that *S. yunnanensis* and *S. nagaensis* are primarily insectiorous and rarely eat plants. This is why the results reflect the food sources and contribution rates of the two nuthatches, even though samples were not collected from all food sources. The insect prey composition of *S. yunnanensis* and *S. nagaensis* overlapped, which is consistent with the results seen for *Sitta tephronota* and *Sitta neumayer*, based on gastrointestinal tract content analysis [5]. Although the insects in the diets of *S. yunnanensis* and *S. nagaensis* were the same at the order level, the proportions of each component differed significantly; *S. yunnanensis* fed predominantly on Orthoptera (78.69%), Hemiptera (7.76%), and Coleoptera (3.96%), while *S. nagaensis* fed mainly on Orthoptera (45.56%), Hemiptera (22.41%), and Hymenoptera (9.56%). These results differ from those for *S. neumayer*, which primarily preyed on Coleopterans, Hemipterans, and Lepidopterans [5]. In our study, Lepidopterans were not identified as a main food for either *S. yunnanensis* or *S. nagaensis* (Table 1), which may be caused by different research methods. Stable isotope analysis mainly reflects the actual intake, digestion, and assimilation information of food, while gastrointestinal content analysis mainly reflects information on food intake. The overlapping of food resources between *S. yunnanensis* and *S. nagaensis* is consistent with the adoption of strategies for food resources observed in other sympatric species. For example, a feather isotope analysis of three sympatric species of closely related seabirds revealed substantial overlap in food resources [21], and Australian snubfin and humpback dolphins that co-exist in the same domain have overlapping dietary resources but different dietary preferences [37].

Based on the theory of competition, *S. yunnanensis* and *S. nagaensis* were hypothesized to exhibit isotopic niche differentiation, enabling them to coexist stably. The isotope niche showed separation between the trophic niches of *S. yunnanensis* and *S. nagaensis* (Figure 4), indicating that differentiation existed in feeding ratios. In addition, *S. yunnanensis* and *S. nagaensis* show differentiation in breeding times in the field; *S. yunnanensis* breeds much earlier than *S. nagaensis* (unpublished data), which may be a reproductive strategy to reduce competition for food resources and improve reproductive success. Niche differentiation plays an important role in the coexistence of closely related species, related species usually have similar functional traits and ecological habits and may exhibit interspecific competition due to niche overlap when resources are limited. The niche differentiation hypothesis predicts that species with niche overlap must show niche differentiation to coexist stably [38], while the niche compensation theory suggests that differentiation must occur in at least one of the three dimensions: time, space, and food [14,39]. In this study, sympatrically coexisting trophic niche differentiation of *S. yunnanensis* and *S. nagaensis* was found to help reduce competition for limited food resources to help achieve coexistence. This is the same feeding strategy as the sympatric harp seal (*Pagophilus groenlandicus*) and ringed seal (*Pusa hispida*); they differ in terms of prey species proportions and size composition, reducing the degree of realized niche overlap [40]. In this study, there was a difference in the percentages of food consumed. This feeding strategy has also been reported in other coexisting animals, in mantled and black howler monkeys in allopatry, their isotopic niches are similar, but the niches of sympatric populations are separated [15]. A study of two giant petrels (*Macronectes* spp.) using stable isotopes and remote sensing tracking techniques found a high degree of overlap in food resources but differentiation in trophic niche and foraging habitat [41], the results of the present study are consistent with this. The evidence of trophic niche partitioning between *S. yunnanensis* and *S. nagaensis* supports the niche differentiation hypothesis.

The food contribution rates of *S. nagaensis* differed substantially between direct observation and stable isotope analysis. Direct observations revealed that the contribution rates of Diptera, Orthoptera, and Lepidoptera were highest, while stable isotope analysis showed that the contribution rates of Orthoptera, Hemiptera, and Hymenoptera were highest. This difference can be explained by methodological differences; stable isotope analyses reflect the food resources assimilated by animals rather than the food consumed by animals [42,43], while the direct observation method records food resources that are consumed. Lepidoptera had the lowest contribution rate in the stable isotope analysis (2.29% for *S. yunnanensis* and 4.12% for *S. nagaensis*). Most Lepidoptera found were small butterflies, moths, or larvae. It has been found that the stable isotope values of animals increase as size increases [19], and the small number of lepidopteran individuals consumed may explain their low contribution rate in the stable isotope analysis.

The δ^15^N isotope niche space between species changes in response to different trophic levels of foraging. In this study, there was no significant difference in δ^15^N between *S. yunnanensis* and *S. nagaensis* (*p* > 0.05), indicating that they were in the same trophic level. However, the trophic niche of *S. yunnanensis* (2.69‰^2^) was larger than that of *S. nagaensis* (0.73‰^2^). The trophic niche breadth occupied by a species in an ecosystem represents its utilization of all available resources, and the isotopic niche breadth varies among species [40]. The larger the width, the stronger the utilization and competition ability of food resources [44,45]. These results indicated that *S. yunnanensis* had stronger utilization and competitive ability, with relatively broad food sources. In a community or ecosystem, differences in feeding habits and habitat among species will lead to differentiation in trophic niches, and overlap in food resources is expected in closely related sympatric species due to the similarity in body structure. Despite this, competition can be reduced through spatial or temporal differentiation in habitat use [46,47]. Future studies of closely related sympatric species should be combined with multi-dimensional explorations of their ecological niches for a more comprehensive understanding of the mechanism underlying their coexistence.

## 5. Conclusions

Sympatric closely related species can reduce competition by allocating resources along spatial, dietary, and temporal axes. In this study, a stable isotope analysis of feathers and potential feeding sources of the sympatric nuthatches *S. yunnanensis* and *S. nagaensis* was performed. The results showed that the two species occupied the same trophic level. In terms of food resources, the two species overlapped and achieved food niche differentiation through differences in feeding ratios, reducing competition and achieving stable coexistence. Compared with *S. nagaensis*, *S. yunnanensis* had better utilization and competitive ability for food resources with a relatively large trophic niche. Ecological niche establishment of sympatric closely related species often involves interspecific interactions across nutritional, temporal, and spatial dimensions. The coexistence of similar species may be regulated through various dimensions, and this process is dynamic. Future research on the coexistence mechanisms of sympatric closely related species should consider integrating multiple dimensions, such as nutritional, temporal, and spatial dimensions, to achieve a more comprehensive understanding of coexistence mechanisms.

## Figures and Tables

**Table 1:** Overview of potential food sources of *S. yunnanensis* and *S. nagaensis* in Yunnan Zixi Mountain. Stable isotope values are presented as means and standard deviations.

Food Source	Sample Size (*n*)	*S. nagaensis* Photograph Number (*n*)	Stable Isotope Signature		Food Contribution Rate (%)	
			δ^13^C‰	δ^15^N‰	*S. yunnanensis* Feather	*S. nagaensis* Feather
Hemiptera	7	3	−25.24 ± 2.35	1.86 ± 2.90	7.76	22.41
Lepidoptera	17	9	−29.48 ± 3.38	2.38 ± 3.19	2.29	4.12
Hymenoptera	4	2	−25.74 ± 0.60	2.89 ± 2.24	3.91	9.56
Coleoptera	12	7	−25.20 ± 3.58	3.33 ± 3.24	3.96	9.30
Diptera	3	13	−24.70 ± 0.63	3.97 ± 1.24	3.36	9.02
Orthoptera	4	11	−23.98 ± 3.46	0.90 ± 0.26	78.69	45.56

**Table 2:** δ^13^C‰ and δ^15^N‰ values and isotopic niche parameters for feather samples from *S. yunnanensis* and *S. nagaensis*.

Isotope	*S. yunnanensis*	*S. nagaensis*
δ^13^C‰	−20.95 ± 0.77	−23.24 ± 0.81
δ^15^N‰	3.67 ± 1.07	3.50 ± 0.54
TA (‰^2^)	6.44	1.73
SEAc (‰^2^)	2.69	0.73

## Data Availability

All data were collected and analyzed by scientific methods in the field and can be provided if necessary.

## Supplementary Materials

The following supporting information can be downloaded at: https://www.mdpi.com/article/10.3390/ani14081146/s1, Table S1: *Sitta yunnanensis* and *Sitta nagaensis* Stable isotopes of food resources; Table S2: Feather stable isotope data of *Sitta yunnanensis* and *Sitta nagaensis*.
