# Supplementary material for: Trophic Niche Differentiation in Two Sympatric Nuthatch Species (Sitta yunnanensis and Sitta nagaensis)
Source: Animals (Basel). 2024 Apr 10;14(8):1146. doi: 10.3390/ani14081146 (PMC11047393; doi:10.3390/ani14081146)
Supplement: Supplementary file 1 [file animals-14-01146-s001.zip › animals-2918863 Supplementary material.pdf]

Table S1: *Sitta yunnanensis* and *Sitta nagaensis* Stable isotopes of food resources

| Sample ID | Species | d13C（‰, PDB） | C content（%） | d15N（‰, Atm-N2） | N content（%） |
|---|---|---|---|---|---|
| 1-1 | Hemiptera | -26.84 | 53.87 | 3.80 | 9.23 |
| 1-2 | Hemiptera | -27.21 | 51.30 | 0.27 | 9.75 |
| 1-3 | Hemiptera | -25.10 | 47.57 | 6.05 | 9.70 |
| 1-4 | Hemiptera | -20.92 | 50.47 | 2.67 | 9.90 |
| 1-5 | Hemiptera | -26.75 | 51.51 | 3.19 | 9.99 |
| 1-6 | Hemiptera | -23.29 | 52.72 | -0.39 | 9.81 |
| 1-7 | Hemiptera | -26.60 | 66.57 | -2.52 | 4.53 |
| 2-1 | Lepidoptera | -31.68 | 64.38 | 1.94 | 6.62 |
| 2-2 | Lepidoptera | -36.21 | 58.85 | 4.26 | 8.48 |
| 2-3 | Lepidoptera | -31.09 | 47.93 | 4.75 | 11.71 |
| 2-4 | Lepidoptera | -25.16 | 47.60 | 2.57 | 11.03 |
| 2-5 | Lepidoptera | -32.23 | 49.19 | 4.90 | 9.70 |
| 2-6 | Lepidoptera | -32.58 | 69.49 | 6.73 | 3.94 |
| 2-7 | Lepidoptera | -30.50 | 62.89 | 3.18 | 7.85 |
| 2-8 | Lepidoptera | -30.37 | 48.47 | 2.40 | 10.94 |
| 2-9 | Lepidoptera | -28.72 | 50.74 | 2.28 | 12.17 |
| 2-10 | Lepidoptera | -29.26 | 53.57 | 4.97 | 10.68 |
| 2-11 | Lepidoptera | -33.12 | 58.02 | 3.92 | 8.50 |
| 2-12 | Lepidoptera | -23.59 | 49.54 | -3.99 | 11.22 |
| 2-13 | Lepidoptera | -24.42 | 48.34 | -4.26 | 11.79 |
| 2-14 | Lepidoptera | -30.06 | 48.23 | -0.49 | 11.00 |
| 2-15 | Lepidoptera | -26.35 | 50.06 | 4.21 | 12.51 |
| 2-16 | Lepidoptera | -26.46 | 51.92 | 4.90 | 10.75 |
| 2-17 | Lepidoptera | -29.44 | 56.39 | -1.66 | 8.36 |

| | | | | | |
|---|---|---|---|---|---|
| 3-1 | Hymenoptera | -25.26 | 49.22 | 2.63 | 11.92 |
| 3-2 | Hymenoptera | -26.58 | 57.84 | 2.16 | 8.40 |
| 3-3 | Hymenoptera | -25.80 | 52.32 | 0.74 | 8.57 |
| 3-4 | Hymenoptera | -25.35 | 50.69 | 6.04 | 10.57 |
| 4-1 | Coleoptera | -22.75 | 59.94 | 1.43 | 7.62 |
| 4-2 | Coleoptera | -23.94 | 51.18 | 2.06 | 9.43 |
| 4-3 | Coleoptera | -17.96 | 55.88 | 11.03 | 7.70 |
| 4-4 | Coleoptera | -27.25 | 50.92 | 6.03 | 10.12 |
| 4-5 | Coleoptera | -28.47 | 47.39 | 1.07 | 11.35 |
| 4-6 | Coleoptera | -27.13 | 49.49 | 2.14 | 9.57 |
| 4-7 | Coleoptera | -32.58 | 50.73 | 1.52 | 9.34 |
| 4-8 | Coleoptera | -26.53 | 51.22 | 1.50 | 9.72 |
| 4-9 | Coleoptera | -23.67 | 48.24 | 4.13 | 12.50 |
| 4-10 | Coleoptera | -24.13 | 49.88 | 7.47 | 10.19 |
| 4-11 | Coleoptera | -24.37 | 49.98 | 1.22 | 10.77 |
| 4-12 | Coleoptera | -23.65 | 49.27 | 0.35 | 10.07 |
| 5-1 | Diptera | -24.76 | 47.78 | 2.58 | 10.60 |
| 5-2 | Diptera | -25.31 | 49.29 | 4.36 | 11.37 |
| 5-3 | Diptera | -24.04 | 49.73 | 4.97 | 9.69 |
| 6-1 | Orthoptera | -25.76 | 49.42 | 1.05 | 10.47 |
| 6-2 | Orthoptera | -18.79 | 48.72 | 0.58 | 9.70 |
| 6-3 | Orthoptera | -25.73 | 52.76 | 0.80 | 9.63 |
| 6-4 | Orthoptera | -25.64 | 47.25 | 1.18 | 12.46 |

Table S2: Feather stable isotope data of *Sitta yunnanensis* and *Sitta nagaensis*

| Sample ID | Species | Acquisition time | d$^{13}$C（‰, PDB） | C content（%） | d$^{15}$N（‰, Atm-$N_2$） | N content（%） |
|---|---|---|---|---|---|---|
| 1-1 | *Sitta yunnanensis* | 2018.12.25 | -21.40 | 44.94 | 4.13 | 12.08 |
| 1-2 | *Sitta yunnanensis* | 2018.12.25 | -20.29 | 45.07 | 4.69 | 12.56 |
| 1-3 | *Sitta yunnanensis* | 2018.12.25 | -21.25 | 44.23 | 4.08 | 12.06 |
| 1-4 | *Sitta yunnanensis* | 2018.12.25 | -21.74 | 45.15 | 5.58 | 13.04 |
| 1-5 | *Sitta yunnanensis* | 2018.12.25 | -20.44 | 44.07 | 4.87 | 13.00 |
| 1-6 | *Sitta yunnanensis* | 2018.12.25 | -20.98 | 44.76 | 4.84 | 12.79 |
| 1-7 | *Sitta yunnanensis* | 2018.12.28 | -21.53 | 46.28 | 2.11 | 12.39 |
| 1-8 | *Sitta yunnanensis* | 2018.12.28 | -21.36 | 44.74 | 2.33 | 12.29 |
| 2-1 | *Sitta yunnanensis* | 2019.05.01 | -21.61 | 45.18 | 5.38 | 13.27 |
| 2-2 | *Sitta yunnanensis* | 2019.05.01 | -21.09 | 44.87 | 5.43 | 13.54 |
| 2-3 | *Sitta yunnanensis* | 2019.05.01 | -19.84 | 44.48 | 2.91 | 12.61 |
| 2-4 | *Sitta yunnanensis* | 2019.05.01 | -21.40 | 44.20 | 2.01 | 12.50 |
| 2-5 | *Sitta yunnanensis* | 2019.05.01 | -20.17 | 46.10 | 3.35 | 12.55 |
| 2-6 | *Sitta yunnanensis* | 2019.05.01 | -20.34 | 45.66 | 3.45 | 13.25 |
| 2-7 | *Sitta yunnanensis* | 2019.05.01 | -20.17 | 45.18 | 3.18 | 12.09 |
| 2-8 | *Sitta yunnanensis* | 2019.05.01 | -20.05 | 46.05 | 3.08 | 12.32 |
| 2-9 | *Sitta yunnanensis* | 2019.05.02 | -20.38 | 45.02 | 3.43 | 12.14 |
| 2-10 | *Sitta yunnanensis* | 2019.05.02 | -19.78 | 45.31 | 3.16 | 12.19 |
| 2-11 | *Sitta yunnanensis* | 2019.05.02 | -21.06 | 45.56 | 3.44 | 12.86 |
| 2-12 | *Sitta yunnanensis* | 2019.05.02 | -21.51 | 44.67 | 3.62 | 12.72 |
| 2-13 | *Sitta yunnanensis* | 2019.05.02 | -22.68 | 44.56 | 3.18 | 12.55 |
| 2-14 | *Sitta yunnanensis* | 2019.05.02 | -21.92 | 43.38 | 2.65 | 12.51 |
| 3-1 | *Sitta nagaensis* | 2018.12.24 | -23.41 | 45.77 | 3.29 | 12.57 |
| 3-2 | *Sitta nagaensis* | 2018.12.24 | -23.49 | 46.00 | 3.78 | 13.16 |

| | | | | | | |
|---|---|---|---|---|---|---|
| 3-3 | *Sitta nagaensis* | 2018.12.24 | -23.21 | 45.91 | 2.86 | 12.49 |
| 3-4 | *Sitta nagaensis* | 2018.12.24 | -21.59 | 46.69 | 4.30 | 13.54 |
| 4-1 | *Sitta nagaensis* | 2019.05.01 | -22.32 | 45.50 | 4.17 | 12.68 |
| 4-2 | *Sitta nagaensis* | 2019.05.01 | -21.89 | 45.17 | 4.58 | 13.24 |
| 4-3 | *Sitta nagaensis* | 2019.05.02 | -23.11 | 45.30 | 3.59 | 12.46 |
| 4-4 | *Sitta nagaensis* | 2019.05.02 | -24.10 | 45.05 | 3.07 | 13.04 |
| 5-1 | *Sitta nagaensis*(baby) | 2019.05.13 | -23.55 | 45.19 | 3.34 | 12.81 |
| 5-2 | *Sitta nagaensis*(baby) | 2019.05.13 | -23.68 | 46.11 | 3.21 | 13.56 |
| 5-3 | *Sitta nagaensis*(baby) | 2019.05.13 | -23.85 | 49.80 | 3.23 | 13.62 |
| 5-4 | *Sitta nagaensis*(baby) | 2019.05.13 | -23.93 | 48.31 | 3.26 | 13.48 |
| 5-5 | *Sitta nagaensis*(baby) | 2019.05.13 | -23.96 | 47.48 | 2.90 | 13.90 |
